# Advances in childhood immunisation in South Africa: where to now? Programme managers’ views and evidence from systematic reviews

**DOI:** 10.1186/1471-2458-12-578

**Published:** 2012-07-31

**Authors:** Charles Shey Wiysonge, Nthombenhle J Ngcobo, Prakash M Jeena, Shabir A Madhi, Barry D Schoub, Anthony Hawkridge, Muki S Shey, Gregory D Hussey

**Affiliations:** 1 Vaccines for Africa Initiative, Institute of Infectious Disease and Molecular Medicine, University of Cape Town, Cape Town, South Africa; 2 Department of Clinical Laboratory Sciences, Division of Medical Microbiology, University of Cape Town, Cape Town, South Africa; 3 Expanded Programme on Immunisation, National Department of Health, Pretoria, South Africa; 4 Department of Paediatrics and Child Health, University of KwaZulu Natal, Durban, South Africa; 5 National Institute for Communicable Diseases, Johannesburg, South Africa; 6 Centre for the AIDS Programme of Research in South Africa (CAPRISA), University of KwaZulu-Natal, Durban, South Africa

## Abstract

**Background:**

The Expanded Programme on Immunisation (EPI) is one of the most powerful and cost-effective public health programmes to improve child survival. We assessed challenges and enablers for the programme in South Africa, as we approach the 2015 deadline for the Millennium Development Goals.

**Methods:**

Between September 2009 and September 2010 we requested national and provincial EPI managers in South Africa to identify key challenges facing EPI, and to propose appropriate solutions. We collated their responses and searched for systematic reviews on the effectiveness of the proposed solutions; in the Health Systems Evidence, Cochrane Library, and PubMed electronic databases. We screened the search outputs, selected systematic reviews, extracted data, and assessed the quality of included reviews (using AMSTAR) and the quality of the evidence (using GRADE) in duplicate; resolving disagreements by discussion and consensus.

**Results:**

Challenges identified by EPI managers were linked to healthcare workers (insufficient knowledge of vaccines and immunisation), the public (anti-immunisation rumours and reluctance from parents), and health system (insufficient financial and human resources). Strategies proposed by managers to overcome the challenges include training, supervision, and audit and feedback; strengthening advocacy and social mobilisation; and sustainable EPI funding schemes, respectively. The findings from reliable systematic reviews indicate that interactive educational meetings, audit and feedback, and supportive supervision improve healthcare worker performance. Structured and interactive communication tools probably increase parents’ understanding of immunisation; and reminders and recall, use of community health workers, conditional cash transfers, and mass media interventions probably increase immunisation coverage. Finally, a national social health insurance scheme is a potential EPI financing mechanism; however, given the absence of high-quality evidence of effects, its implementation should be pilot-tested and the impacts and costs rigorously monitored.

**Conclusion:**

In line with the Millennium Development Goals, we have to ensure that our children’s right to health, development and survival is respected, protected and promoted. EPI is central to this vision. We found numerous promising strategies for improving EPI performance in South Africa. However, their implementation would need to be tailored to local circumstances and accompanied by high-quality monitoring and evaluation. The strength of our approach comes from having a strong framework for interventions before looking for systematic reviews. Without a framework, we would have been driven by what reviews have been done and what is easily researchable; rather than the values and preferences of key immunisation stakeholders.

## Background

The Expanded Programme on Immunisation (EPI), which is one of the most powerful and cost-effective public health programmes to improve child survival [1-4], was introduced in South Africa in 1974. The programme, however, remained fragmented because of the system of apartheid until 1995; when the national EPI was formed through the unification of all immunisation services in the country [5]. Since then, there have been significant advances in immunisation services delivery in South Africa, including the introduction of the hepatitis B virus vaccine and *Haemophilusinfluenzae* type b conjugate vaccine into EPI in 1995 and 1999, respectively. In addition, neonatal tetanus elimination was validated in 2002 and interruption of wild poliovirus transmission was declared in 2006 [5]. More recently, in April 2009, South Africa became the first country in Africa to introduce nationwide routine childhood vaccination against rotavirus and *Streptococcus pneumoniae*; at no cost to recipients.

Despite these advances, there is some evidence that EPI South Africa faces a number of challenges [6-9]. The vaccination coverage is low (with only two-thirds of children estimated to receive the full series of three doses of the diphtheria-tetanus-pertussis vaccine by one year of age) [8,10], measles outbreaks are frequent [6,9], and community knowledge of immunisation is low [7]. In order to ensure evidence-informed selection and implementation of effective healthcare interventions [11] that would overcome existing barriers and reach every child in South Africa with life-saving vaccines, we conducted an audit to elicit programme managers’ views of the challenges facing EPI and potential solutions for these challenges. Our objectives were: (1) to determine what EPI programme managers in South Africa consider as key barriers to effective implementation of the programme in the country; (2) to determine what EPI programme managers consider as the best interventions to overcome the identified barriers; and (3) to conduct a comprehensive search of peer-reviewed literature, identify, and synthesise current best evidence on the effectiveness of the interventions proposed by the EPI programme managers.

## Methods

South Africa has one national and nine provincial (sub-national) EPI managers. In September 2009, we sent e-mails to the national EPI manager and the nine provincial EPI managers, requesting the managers to identify key challenges facing EPI. The two questions asked were:

1. What, in your opinion, are the five key challenges to childhood immunisation in South Africa (List the challenges from the most important challenge to the least important)?

2. What would be the solutions, in your opinion, to these challenges (Only one solution for each challenge)?

Eight days after the initial email request, we sent a follow-up reminder to non-responders. Responses were collated two weeks later. The findings of this first audit were presented at a national immunisation conference in October 2009; which was attended by all key immunisation stakeholders in South Africa [12]. The conference agenda included a critical appraisal of vaccines and vaccine administration, globally and with specific reference to South Africa. In June 2010, the two questions were again sent to all the 10 EPI managers in the country.

The exercise was initially undertaken as a programme evaluation for management purposes, and therefore no ethics review board approval was sought. However, only later we realised that the results could be of interest to a wider audience. All EPI managers who responded to the questionnaire provided consent for their responses to be published. In addition, we obtained the permission of the South African National Department of Health to publish the findings. In this paper we present the key challenges identified by the managers, the key solutions they proposed, and the findings from systematic reviews on the effects of the proposed interventions. Each respondent provided five barriers and five corresponding corrective interventions. In summarising the responses, we treated the barriers identified (five per manager) equally without applying any weighting; irrespective of whether the barriers were identified by the national or provincial managers, or whether the barrier was classified by the manager as most important or least important. We did the same for the proposed interventions (one per identified barrier).

**Figure 1:**
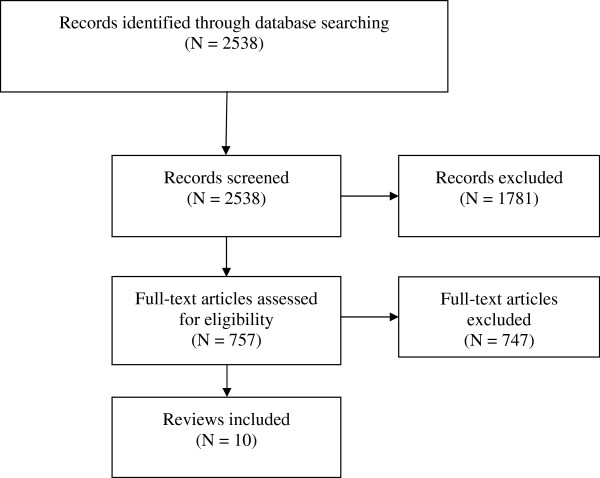
Flow diagram showing the search and selection of reviews.

On 30 November 2010 we searched the Health Systems Evidence database, the Cochrane Database of Systematic Reviews, the Database of Abstracts of Reviews of Effectiveness, and PubMed. One author (CSW) conducted the search using the search strategy shown in Table 1. We screened the search outputs in the order in which the databases are listed here, starting with Health Systems Evidence database and ending with PubMed. Figure 1 shows a summary of the search and selection process. When we found more than one systematic review that assessed a particular intervention, we chose the one that was more comprehensive and/or more recent. Two authors (CSW and MSS) independently screened the search results, selected relevant systematic reviews, and assessed the quality of selected reviews using the AMSTAR tool [13]. In particular, we assessed whether the authors of the review reported the study selection criteria; conducted a literature search that was comprehensive enough to avoid publication, language and indexing biases; undertook duplicate study selection and data extraction; used reliable criteria to assess the risk of bias in included studies; reported the characteristics of included studies appropriately; and combined data from included studies using reliable methods. Based on these criteria, we concluded whether the review was well conducted (i.e. reliable) or not. We have only reported data from reviews that we considered to be reliable. At each stage, the two authors compared their results and resolved any disagreements by discussion and consensus.

**Table 1:** Search strategy for identification of eligible reviews

***A. Interventions directed at healthcare workers***	
**Health Systems Evidence:**	
Health system topic	Provider-targeted strategy
Type of synthesis	Systematic review OR policy brief OR systematic review protocol
Type of question	Effectiveness
Publication date range	2000 to 2010
**Cochrane Database of Systematic Reviews (CDSR):**	
Search all text	(Training OR education OR workshop OR supervision OR (outreach AND visit*) OR (audit AND feedback) OR monitoring) AND (“Immunization”[Mesh] OR “Vaccination”[Mesh] OR “ Immunization Programs”[Mesh])
Limits	None
**Database of Abstracts of Reviews of Effectiveness (DARE):**	
Search all text	(Training OR education OR workshop OR supervision OR (outreach AND visit*) OR (audit AND feedback) OR monitoring) AND (“Immunization”[Mesh] OR “Vaccination”[Mesh] OR “Immunization Programs”[Mesh])
Limits	None
**PubMed:**	
Search terms	(Training OR education OR workshop OR supervision OR (outreach AND visit*) OR (audit AND feedback) OR monitoring) AND (“Immunization”[Mesh] OR “Vaccination”[Mesh] OR “Immunization Programs”[Mesh])
Publication date	01 January 2000 to 31 December 2010
Publication type	Reviews
***B. Interventions directed at parents or communities***	
**Health Systems Evidence:**	
Health system topic	Consumer-directed strategy
Type of synthesis	Systematic review OR policy brief OR systematic review protocol
Type of question	Effectiveness
Publication date range	2000 to 2010
**Cochrane Database of Systematic Reviews (CDSR):**	
Search all text	(mobiliz* OR mobilis* OR communicat* OR advoca*) AND (“Immunization”[Mesh] OR “Vaccination”[Mesh] OR “Immunization Programs”[Mesh])
Limits	None

Finally, we used the GRADE approach [14,15] to assess the quality of the evidence for the effectiveness of the proposed strategies. This method results in an assessment of the quality of a body of evidence as high, moderate, low, or very low. High quality evidence implies that “further research is very unlikely to change our confidence in the estimate of effect”. Moderate quality evidence means that “further research is likely to have an important impact on our confidence in the estimate of effect and may change the estimate”. Evidence is considered of low quality if “further research is very likely to have an important impact on our confidence in the estimate of effect and is likely to change the estimate”, and very low quality if “we have very little confidence in the effect estimate”. We began the rating of the quality of evidence with the study design; evidence from systematic reviews of randomised controlled trials as high-quality and that from systematic reviews of observational studies as low-quality. In addition, five reasons led us to downgrade the quality of evidence from systematic reviews of randomised controlled trials and three to upgrade the quality of evidence from systematic reviews of observational studies. For pooled data from randomised controlled trials, the factors that led to rating down the quality of evidence were risk of bias, heterogeneity, indirectness, imprecision, and publication bias. Regarding risk of bias, concerns that limited our confidence in the evidence include lack of allocation concealment, lack of blinding of outcome assessment, and a large loss to follow-up. Heterogeneity of effects across studies for which there were no compelling explanations also reduced our confidence in the evidence. Indirectness refers to differences between the population, intervention, comparison group and outcome of interest to us, and those included in the relevant reviews e.g. we used the evidence on strategies for improving patients’ understanding of health information as a proxy for evidence on parents’ understanding of the importance of childhood immunisation [16]. For imprecision, if we found that studies included relatively few participants and few events and thus had estimates of effects with wide confidence intervals, we rated down the quality of the evidence. Finally, we downgraded the quality of evidence if there was a high likelihood of publication bias. Furthermore, we upgraded the quality of the evidence if the pooled estimates revealed a large magnitude of effect, if we had negligible concerns about confounders, or if there was a strong dose-response gradient [14].

## Results

We received responses from the national EPI manager and five provincial EPI managers in the first round (giving a total of 6 x 5 = 30 identified challenges). For the second round, we received responses from the six EPI managers who sent responses in the first round plus three additional provincial managers (i.e. a total of 9 x 5 = 45 challenges). One provincial manager did not respond. Table 2 shows that consolidation of the responses from the first round revealed the five key EPI challenges to be (in descending order): insufficient knowledge of vaccines and immunisation among staff; staff shortages and high staff turn-over; financial constraints; poor communication among stakeholders (including insufficient advocacy and insufficient social mobilisation); and sub-optimal collaboration between the public and private health sectors. The second round revealed the first four key challenges to be identical to those of the first round; as shown in Table 3. Rumours and reluctance from parents, as well as sub-optimal collaboration between the public and private health sectors occupied fifth position.

**Table 2:** Summary of responses from first round

	**Barrier**	**Frequency**	
		**Count**	**Proportion**
1.	Insufficient knowledge of vaccines and EPI practices among staff	13	43.3%
2.	Financial constraints	7	23.3%
3.	Staff shortages and high staff turn-over	7	23.30%
4.	Poor communication among stakeholders	2	6.7%
5.	Challenges working with the private sector	2	6.7%
	**Total**	**30**	**100.0%**

**Table 3:** Summary of responses from second round

	**Barrier**	**Frequency**	
		**Count**	**Proportion**
1.	Insufficient knowledge of vaccines and EPI practices among staff	14	31.1%
2.	Staff shortages and high staff turn-over	9	20.0%
3.	Financial constraints	8	17.8%
4.	Poor communication among stakeholders	6	13.3%
5.	Challenges working with the private sector	3	6.7%
6.	Resistance from parents and anti-immunisation rumours	3	6.7%
7.	Vaccine stock-outs	2	4.4%
	**Total**	**45**	**100.0%**

The strategies proposed by the EPI managers for addressing the EPI challenges are highlighted in Table 4. For example, the managers proposed training, supportive supervision, and audit and feedback for addressing the insufficient knowledge of immunisation issues among healthcare workers. Through a comprehensive search and selection process (as shown in Figure 1), we identified many well-conducted systematic reviews on strategies for improving the performance of healthcare workers; including educational meetings [17], supportive supervision or educational outreach i.e. “a personal visit by a trained person to health workers in their own settings”[18], audit and feedback i.e. “a summary of performance over a specified period of time given in a verbal or written format”[19], and printed educational materials [20]. Concerning strategies for strengthening vaccine advocacy and social mobilisation, we found reliable reviews on parent reminder and recall systems [21], use of community health workers [22], interactive communication tools [16], mass media interventions [23], and conditional cash transfers i.e. monetary transfers made to disadvantaged households on the condition that they comply with some pre-determined requirements in relation to health care, such as vaccinating their children [24]. Finally, regarding appropriate EPI funding schemes that would address the identified financial and human resource constraints, we found a comprehensive systematic review on the impacts of using social health insurance schemes to finance health care [25]. We provide a description of the objectives, type and number of studies included, participants, settings, interventions, and outcomes assessed in the included systematic reviews in Table 5.

**Table 4:** Strategies proposed by EPI managers for addressing barriers

	**Barrier**	**Proposed solution**
1	Insufficient knowledge of vaccines and immunisation	Training, supportive supervision, and audit and feedback
2	Financial constraints	Government should make appropriate financial arrangements for financing the EPI
3	Staff shortages and high staff turn-over	Government should put in place appropriate strategies for recruitment and retention of staff
4	Poor communication among stakeholders	Use best available evidence for vaccine advocacy and social mobilisation
5.	Resistance from parents and anti-immunisation rumours	Strengthen social mobilisation and provide timely evidence-based response to rumours

**Table 5:** Characteristics of included systematic reviews

**Review question / objective**	**Data sources**	**Interventions**	**Data collection and analysis**
Reference [16]			
What are the most effective communication tools to improve patient understanding of ‘evidence’?	Cochrane Library, Medline, Psychinfo, Embase, Cancerlit, authors’ personal files.	10 SRs and 17 RCTs on tailored print information, decision aids, consultation summaries or instructions (audiotapes, written and verbal), provider training in a patient-centred approach with or without risk communication, video, interactive computer aids/touch screens, evidence-based leaflets, and question prompts.	Conducted duplicate study selection, critical appraisal (of RCTs and SRs using validated checklists) and data extraction; and narrative synthesis and meta-analysis, as appropriate.
	Last search: June 2004		
Reference [17]			
Are educational meetings and workshops effective in improving professional practice or healthcare outcomes?	EPOC trials register, EMBASE, reference lists	81 RCTs of educational meetings (alone or as part of multifaceted interventions); conducted in North America (31 studies), Europe (34), Australia and New Zealand (4), South East Asia (4), Latin America (3), and sub-Saharan Africa (4).	Conducted duplicate study selection, critical appraisal (of RCTs and SRs using validated checklists) and data extraction; and narrative synthesis and meta-analysis, as appropriate.
	Last search: March 2006.		
Reference [18]			
Are educational outreach visits effective in improving health professional practice and healthcare outcomes?	EPOC register, Medline, EMBASE, reference lists	69 RCTs of educational outreach visits (alone or as part of multifaceted interventions); conducted in North America (23 RCTs), Europe (36), Australia (8), and South East Asia (3).	Conducted duplicate study selection, critical appraisal (of RCTs and SRs using validated checklists) and data extraction; and narrative synthesis and meta-analysis, as appropriate.
	Last search: March 2007.		
Reference [19]			
Is audit and feedback effective in improving professional practice and health care outcomes?	EPOC register, Medline, EMBASE, reference lists	118 RCTs of audit and feedback (alone or as part of multifaceted interventions); conducted in North America (67), Europe (30), Australia (9), South East Asia (3), and sub-Saharan Africa (1).	Conducted duplicate study selection, critical appraisal (of RCTs and SRs using validated checklists) and data extraction; and narrative synthesis and meta-analysis, as appropriate.
	Last search: February 2006		
Reference [20]			
To determine the effectiveness of printed educational materials in improving process outcomes and patient outcomes.	EPOC register, Medline, EMBASE, CENTRAL, DARE, CINAHL, CAB Health, reference lists.	12 RCTs, 1 CBA, and 10 ITS of printed educational materials; conducted in North America (14) and Europe (9).	Conducted duplicate study selection, critical appraisal (of RCTs and SRs using validated checklists) and data extraction; and narrative synthesis and meta-analysis, as appropriate.
	Last search: March 2007		
Reference [21]			
To assess the overall effectiveness of patient reminder or recall systems, or both, in improving immunisation coverage	EPOC register, Medline, EMBASE, CENTRAL, PsychINFO, CINAHL, Sociological Abstracts, CAB Abstracts, reference lists.	40 RCTs and 3CBAs of of patient reminder or recall systems; conducted in North America (37), Europe (2) and Australia and New Zealand (4)	Conducted duplicate study selection, critical appraisal (of RCTs and SRs using validated checklists) and data extraction; and narrative synthesis and meta-analysis, as appropriate.
	Last search: May 2007		
Reference [22]			
To assess the effects of lay health worker interventions in primary and community health care on maternal and child health and the management of infectious diseases.	Medline, EMBASE, CENTRAL, PsychINFO, CINAHL, Sociological Abstracts, CAB Abstracts, British Nursing Index and Archive, POPLINE, WHOLIS, ISI Web of Science, Healthstar, reference lists.	82 RCTs on use of lay health worker interventions; conducted in high-income countries (55), middle-income countries (12), and low-income countries (15).	Conducted duplicate study selection, critical appraisal (of RCTs and SRs using validated checklists) and data extraction; and narrative synthesis and meta-analysis, as appropriate.
	Last search: February 2010		
Reference [23]			
To assess the effect mass media interventions on utilisation of health services.	EPOC register, Medline, EMBASE, Eric, PsycLit, hand search of relevant journals, reference lists.	20 ITS and 1 CBA of mass media campaigns (using radio, television, newspapers, posters and leaflets); conducted in Europe (10), North America (5), Australia (4), and Latin America (1).	Conducted duplicate study selection, critical appraisal (of RCTs and SRs using validated checklists) and data extraction; and narrative synthesis and meta-analysis, as appropriate.
	Last search: 1999		
Reference [24]			
To assess the effectiveness of conditional monetary transfers in improving access to care and health outcomes, in particular for poorer populations in LMICs.	EPOC register, CENTRAL, Medline, EMBASE, Popline, CAB-Direct, WHOLIS, LILACS, many other databases and websites/online resources, reference lists.	4 RCTs and 2 CBAs of conditional cash transfer programmes; conducted in Latin America (5) and sub-Saharan Africa (1).	Conducted duplicate study selection, critical appraisal (of RCTs and SRs using validated checklists) and data extraction; and narrative synthesis and meta-analysis, as appropriate.
	Last search: May 2009.		
Reference [25]			
To assess the effectiveness of risk protection mechanisms in improving access to care in LMICs	EPOC register, CENTRAL, Medline, EMBASE, Popline, CAB-Direct, WHOLIS, LILACS, many other databases and websites/online resources, reference lists.	Authors found few reports of social health insurance schemes operating at national level in LMICs; but none of these studies was an RCT, CBA, or ITS.	Duplicate screening of search output and assessment of potentially eligible studies for inclusion.
	Last search: May 2009.		

*EPOC* Cochrane Effective Practice and Organisation of Care Group, *SR* systematic review, *RCT* randomised controlled trial; controlled before-and-after study, *ITS* interrupted time series analyses, *CENTRAL* Cochrane Central Register of Controlled Trials, *DARE* Database of Abstracts of Reviews of Effectiveness, *LMICs* low and middle-income countries.

The rating of the quality of currently available evidence on the proposed remedial strategies is shown in Table 6. There exists high-quality evidence [14] showing that interactive educational meetings, audit and feedback, and supportive supervision can improve healthcare worker performance [17-19]. The evidence on the effectiveness of passive distribution of printed educational materials [20] in improving healthcare worker performance is of low quality. Regarding strategies aimed at increasing community demand and support for immunisation services, moderate-quality evidence shows that parent reminder and recall systems [21], use of community health workers [22], mass media interventions [23], and conditional cash transfers [24] may increase routine immunisation coverage. There also exists moderate-quality evidence that structured and interactive communication tools may increase parents’ understanding of the importance of childhood immunisation [16]. Finally, there currently exists very low quality evidence showing that a social health insurance scheme may improve the use of health services in low and middle-income countries [25].

**Table 6:** Summary of the quality of evidence

**Challenges identified by EPI managers**	**Remedial strategies suggested by EPI managers**	GRADE quality of evidence*
Insufficient knowledge of vaccines and immunisation issues among health workers	Regular education	High
	Supportive supervision/educational outreach	High
	Audit and feedback	High
	Printed educational materials.	Low
Anti-immunisation rumours and resistance from parents	Parent reminder and recall systems	Moderate
	Community health workers	Moderate
	Mass media	Moderate
	Structured, tailored, or interactive communication tools	Moderate
	Conditional cash transfers	Moderate
Insufficient financial and human resources	Tax-funded financing of immunisation programmes	No systematic review of effects
	Social health insurance scheme for financing of EPI	Low

*GRADE quality of evidence (From reference [15]):

High quality: “We are very confident that the true effect lies close to that of the estimate of the effect”.

Moderate quality: “We are moderately confident in the effect estimate: The true effect is likely to be close to the estimate of the effect, but there is a possibility that it is substantially different”.

Low quality: “Our confidence in the effect estimate is limited: The true effect may be substantially different from the estimate of the effect”; Very low quality: “We have very little confidence in the effect estimate: The true effect is likely to be substantially different from the estimate of effect”.

## Discussion

Decision makers in South Africa need to use considerable judgement about how best to use limited resources they have for maintaining and improving the quality of health care in order to maximise population benefits. In making such decisions, they need to consider the potential areas for quality improvement activities, the likely benefits and costs required to introduce new quality improvement interventions, and the likely benefits and costs as a result of any changes in the behaviour of healthcare workers. With the values and preferences of programme managers as a starting point, we found that the use of interactive educational meetings and workshops, audit and feedback, supportive supervision, parent reminder and recall systems, conditional cash transfers, community health workers, mass media interventions, and interactive communication tools could be effective in improving EPI performance in South Africa. In selecting which combination of interventions to use for which community and at which time point, decision makers would need to consider population characteristics, available resources, and competing priorities. A major strength of our approach comes from having a framework for interventions (i.e. collating managers’ views of the barriers to effective implementation of the EPI programme and their proposals of effective interventions for addressing these barriers) before looking for systematic reviews of effects. If we had searched the literature for effective interventions for improving childhood immunisation, without such a framework, we would have been driven by what systematic reviews have been done and what is easily researchable; rather than the priorities of key immunisation stakeholders.

The settings and designs of the studies included in the systematic reviews that assessed the effects of interactive educational meetings, audit and feedback, and educational outreach visits for improving healthcare worker performance varied widely; but the studies consistently showed that these strategies can improve healthcare worker performance [17-19].The reviews also found that multifaceted interventions may not be any more effective than educational meetings, outreach visits, or audit and feedback alone [17-19]. The consistency of effects across different study designs and healthcare settings and conditions suggests that these findings would be applicable to the EPI in South Africa. The low quality of the evidence on the effects of passive distribution of printed educational materials [20] implies that we have limited confidence in the effectiveness of this strategy to improve the knowledge of vaccine and immunisation issues among healthcare workers in South Africa [14]. Social mobilisation for immunisation may include active community participation, contextualisation of information in the local customs and culture, and involvement of a broad range of stakeholders and the mass media. The moderate quality of the evidence on parent reminder and recall systems [21], community health workers [22], interactive communication tools [16], conditional cash transfers [24], and mass media interventions [23] is an indication that these strategies could have significant effects in mobilising communities and increasing demand for routine childhood immunisation services in South Africa.

Appropriate financing mechanisms are needed to ensure that there are sufficient funds (at all times in all health districts) to recruit and retain a sufficient number of qualified staff, procure adequate quantities of vaccines, purchase and maintain the appropriate vaccine-related equipment and other logistics; and maintain and improve the quality of care. Evidence of effects, population characteristics, societal values, and attitudes are important when selecting funding schemes which aim to ensure universal coverage of health services. Childhood immunisation services are provided free of charge at public health facilities in South Africa [5]. Therefore, sustainable EPI financing mechanisms relevant to the South African context would include the use of general tax revenues or a social health insurance scheme to cover the costs of immunisation services. Social health insurance refers to compulsory health insurance that aims to provide universal coverage. The compulsory nature of social health insurance should reduce adverse selection and enable redistributive mechanisms between poor and affluent segments of the population. However, few examples exist of social health insurance schemes operating at a large scale in low and middle-income countries; none of which provides strong evidence related to their impact [25]. The National Health Insurance (NHI), whose planned introduction in the health sector in South Africa has become a very topical issue, is a type of social health insurance scheme. The South African National Department of Health indicates that the NHI “is based on the key principles of universality, that every South African would be entitled to benefit from the services it covers, and that it would be funded partly by compulsory contributions by all persons who are earning an income and partly by tax. All these funds would be placed in a single pool. This pool would be available to fund all health care in the public and private health sector under conditions that would apply to all health care service providers.” [26].

Our study has some limitations. One provincial manager did not respond to our questions. However, given the consistency of the key challenges across the other eight provinces, we do not think that the EPI challenges in the province of the manager who did not respond would be substantially different from those of the other provinces. There is a possibility that the presentation of findings of the first round to EPI managers may have influenced the responses in the second round of the audit. However, this does not seem to have been the case because new challenges (e.g. reluctance from parents) emerged from the second round. Regardless of these potential shortcomings, we believe that this study is a good reflection of the challenges encountered in the planning, delivering, and monitoring of childhood immunisation services in South Africa. Most of the evidence of effects described above comes from studies conducted in high-income countries, and applicability to South Africa or other low and middle-income countries may be limited. There is thus a need for high-quality studies from low and middle-income countries, assessing the effects of strategies for improving immunisation services. In the meantime, implementation of such strategies in South Africa should be pilot-tested and their impacts and costs rigorously monitored and evaluated [11].

## Conclusion

In line with the Millennium Development Goals, we have to ensure that our children’s right to health, development and survival is respected, protected and promoted. EPI is central to this vision. We found numerous promising strategies for improving EPI performance in South Africa. However, their implementation would need to be tailored to local circumstances and accompanied by rigorous monitoring and evaluation. The suggested interventions (by EPI programme managers) for handling immunisation challenges and the availability (or lack) of sound scientific evidence on their effectiveness, emphasise the need for partnerships between health policy makers, programme managers, and researchers in order to ensure that health decisions are always informed by the best available evidence [27,28]. Collaboration between the three stakeholders needs to be continuous, as the results of this audit show that challenges to immunisation may vary from time to time. Such policymaker-implementer-researcher partnerships are needed to create a health system that is effective, equitable, and sustainable; one in which EPI will be better able to meet its obligations.

## Competing interests

The authors declare that they have no competing interests.

## Authors’ contribution

CSW and GDH conceived the study, CSW and MSS collected and analysed the data, and CSW wrote the first draft of the paper. CSW, NJN, PMJ, SAM, BDS, AH, MSS and GDH made significant contributions to the interpretation of the data, and revision of the manuscript. All authors approved the final version for publication.

## Pre-publication history

The pre-publication history for this paper can be accessed here:

http://www.biomedcentral.com/1471-2458/12/578/prepub
